# Commentary: Quantifying the Human Mortality Costs of Patent-Based Intellectual Property: How Many Premature Deaths are Due to Patents?

**DOI:** 10.1007/s10728-026-00585-y

**Published:** 2026-07-24

**Authors:** Kristina M. L. Acri née Lybecker

**Affiliations:** Gerald L. Schlessman Professor of Economics, Colorado College, Colorado Springs, CO USA

## Abstract

This comment reviews he article, “Quantifying the HumanMortality Costs of Patent-based Intellectual Property: How Many PrematureDeaths are due to Patents?”, by Joshua M. Pearce and argues that the analysisis overly simplistic.While theaffordability of medicine is a troubling challenge, one must examine thebenefits of intellectual property protection as well as the costs in order toprovide meaningful analysis.By failingto do so the author does us all a disservice by blaming the IP system, anobvious target.The truth is far morenuanced and serious readers deserve an analysis that reflects this and shedslight on the subtleties of the problem rather than turning to an easyscapegoat.

Keywords: intellectual property, patents, insulin

The article, “Quantifying the Human Mortality Costs of Patent-based Intellectual Property: How Many Premature Deaths are due to Patents?”, by Joshua M. Pearce asserts that patents result in price increases that inhibit access to medicines (specifically, insulin) which results in premature deaths. While the affordability of medicine is a troubling problem, it is overly simplistic to analyze the cost of patent-based intellectual property without also measuring the benefits. Specifically, the paper stops short of using the necessary methodology to answer the research question with validity. Accounting for all necessary variables would likely result in the opposite conclusion: that patents have saved the lives of diabetics in aggregate, pose no barrier to insulin access in the US compared to Canada, and allow Americans to access the latest insulin technologies at a much greater rate than Canadians.

The author claims to find that insulin-related patents result in over 94,000 American premature deaths annually. However, the author readily acknowledges that “[b]efore insulin, diabetes was a death sentence”. Accordingly, the technological advances protected by insulin-related patents are thereby responsible for maintaining, if not saving, the lives of the 38 million Americans with diabetes [1]. While premature deaths suggest a much more dramatic conclusion, the article provides no discussion of the lives saved by patented medicines, or the number of medicines in existence because of the incentives provided by the patent system.

The article’s methodology also fails to account for numerous alternative explanations. The author essentially argues, and purports to test, that the difference in the death rates of diabetics in the U.S. and Canada can be solely traced to the higher price of insulin in the U.S. which is a result of patents. While the author acknowledges several confounding factors, the methodology accounts for none of them, including actual drug prices, the availability of universal healthcare in Canada, obesity rates in the U.S. that are almost double those in Canada, and the lack of detailed cause of death data. Questions like those posed in this paper should be investigated with rigorous empirical testing, based on econometric regression analysis, and a large sample of quality data. Moreover, it is essential to include control variables in order to isolate the hypothesized effect. Without rigorous analysis, it is impossible to draw the conclusions described in this paper.

Fundamentally, the author argues that lives lost are due to a lack of access to insulin because of high prices resulting from patent protection. However, the paper fails to provide evidence of any of the links in this logical chain.

- It does not establish that these lives were lost because the individuals did not take insulin.
- It does not establish that these diabetics did not take insulin due to high prices.
- It does not establish that the price of insulin is too high due to patent protection.

The claims asserted in this study should be based in thorough research, rigorous testing, and conclusive evidence. Alternative explanations must be investigated and systematically eliminated. The study falls short on all counts.

Consider, for example, the alternate hypothesis that the high price of insulin is the result of “pharmacy benefit managers...inflating insulin prices and steering patients toward higher-cost insulin products to increase their profits”, as charged by the Federal Trade Commission and reported by the New York Times [2]. One must also consider that for years, the U.S. Food and Drug Administration lacked a regulatory pathway that would allow companies to develop and sell lower-priced versions of insulin, as noted in the Journal of Diabetes Science and Technology [3]. And perhaps the prices are subject to federal policies that regulate drug prices that may have unintended consequences as described more recently by the New York Times [4]. The author seemingly did not consider alternative explanations and provides no discussion or evidence that such explanations were evaluated and eliminated as the cause of high insulin prices.

It is also important to recognize that the selection of insulin is a poor choice to “Quantifying the Human Mortality Costs of Patent-based Intellectual Property”. Insulin is more an exception-to-the-rule than an exemplary case study. First, “insulin” is not a single drug, does not embody a single technology, and is not produced by a single monopolist. Banting and Best’s discovery of insulin 100 years ago was a far cry from the products that diabetics utilize today. The author describes the technological advances that benefit today’s patients as “minor developments in the production and delivery of insulin”. Those advances were critical advances made by dedicated scientists, not minor adjustments: the capacity for large-scale production of insulin, slower-acting insulins, genetically engineered synthetic “human” insulin, ultra-rapid insulin, ultra-long acting insulin, pens and pumps [5]. It is overly simplistic to use the single term “insulin” to describe the original refined insulin from the pancreases of cattle to the biosynthetic human insulin available today. Rather than a single discovery, present-day insulin evolved from an unceasing flow of remarkable innovations. Each of these innovations is responsible for an additional leap in the level of patient adherence which countless studies document as improving length and quality of life.

It is also important to note that an exploration of antidiabetic drugs in the U.S. and Canada reveals that the same drugs are used in both countries. Specifically, analog insulin use predominates in both nations at similar rates (approximately 80%) [6, 7]. Access to the same forms of insulin at the same rate suggests that neither patents nor prices create an access issue for patients. In reality, patent protection should provide American patients with faster access to newer/better treatments. Given this, it is possible that more Canadians die due to the lack of access to newer/better drugs.

Finally, it is important to recognize that adherence to prescription regimes is a significant problem globally and independent of patents. In a systematic review and meta-analysis of the prevalence of adherence to insulin therapy in patients with diabetes, researchers found the overall estimated prevalence of adherence to insulin therapy in both types of diabetes was 55.37% [8]. According to the World Health Organization (WHO) report on chronic diseases that require long-term treatment, adherence was found to be around 50% in developed countries and much lower in developing countries [9]. The author does not establish that the lack of adherence in the United States is any different from what is seen in countries where patents are not an issue. There is no evidence that there is a loss of life because patients are unable to afford insulin or that they are unable to afford insulin because of high prices due to patent protection.

The journal’s commitment is admirable, promoting dialogue and debate to illustrate the importance of conceptual and normative analysis to health policy, practice and research. This article, however, undermines that objective. While the affordability of insulin and other medicines is a serious and concerning issue, the author does us all a disservice by blaming the IP system, an obvious target by any standard. The truth is far more nuanced and serious readers deserve an analysis that reflects this and sheds light on the subtleties of the problem rather than turning to an easy scapegoat.
